# Causal relationships between somatic movement, brain structures, and mental well-being: A multi-stage Mendelian randomization study

**DOI:** 10.1017/S0033291726104097

**Published:** 2026-04-10

**Authors:** Lixin Guo, Yuhao Shen, Jie Wen, Kaijie An, Dan Zhang, Xufeng Zhao, Wenwei Zhang, Jiajia Zhu, Yinfeng Qian

**Affiliations:** 1 Department of Radiology, The First Affiliated Hospital of Anhui Medical University, Hefei, China; 2 Research Center of Clinical Medical Imaging, Anhui Province, Hefei, China; 3 Anhui Provincial Key Laboratory for Brain Bank Construction and Resource Utilization, Hefei, China

**Keywords:** brain structure, Mendelian randomization, mental well-being, physical activity, sedentary behavior

## Abstract

**Background:**

While the relationships between somatic movement, mental well-being, and brain health have been well established, the causal nature and underlying mechanisms of such associations remain incompletely understood.

**Methods:**

By applying multi-stage Mendelian randomization to multi-source summary data derived from genome-wide association studies, we examined the causal effects of 4 somatic movement measures on 2 mental well-being indices and 13 types of brain structures, followed by testing the mediating roles of brain structures in accounting for the causal associations between somatic movement and mental well-being.

**Results:**

Two-sample Mendelian randomization revealed that more physical activity was causally associated with greater mental well-being (life satisfaction and positive affect), while more sedentary behavior (longer leisure screen time and more sedentary behavior at work) with lower mental well-being. With respect to brain structures, sedentary behavior was causally linked to decreased volume, surface area, and local gyrification index in distributed cortical regions. Remarkably, decreased surface area of the piriform cortex was found to mediate the causal associations between sedentary behavior and lower mental well-being.

**Conclusions:**

Our findings not only complement and extend earlier reports on the associations of somatic movement with mental well-being and brain health by further resolving the causality but also help elucidate the neural mechanisms by which sedentary behavior adversely affects mental well-being.

## Introduction

Somatic movement, encompassing physical activity and sedentary behavior, is a pivotal lifestyle factor that is well known to influence mental well-being. Physical activity has been generally recognized for its positive impact on mental well-being, including reduction of depressive (Schuch et al., 2018) and anxiety symptoms (Schuch et al., 2019), improvement of cognitive function (Erickson et al., 2019; Feng et al., 2024; Hötting & Röder, 2013), enhancement of emotional well-being, and elevation of subjective life satisfaction (Buecker, Simacek, Ingwersen, Terwiel, & Simonsmeier, 2020). In contrast, sedentary behavior has frequently been shown to exert detrimental effects on mental well-being, including increased anxiety symptoms (Kandola, Lewis, Osborn, Stubbs, & Hayes, 2020) as well as an increased risk of cognitive decline (Falck, Davis, & Liu-Ambrose, 2017) and depression (Hoare, Milton, Foster, & Allender, 2016; Huang et al., 2020; Zhai, Zhang, & Zhang, 2015).

The associations between somatic movement and brain health have long been a topic of active investigation in neuroscience. For example, an extensive body of neuroimaging research has established the intimate links between physical activity and greater brain structural features including gray matter volume, cortical thickness, and white matter integrity (Domingos, Pêgo, & Santos, 2021; Erickson, Hillman, & Kramer, 2015; Erickson, Leckie, & Weinstein, 2014; Firth et al., 2018; Fox et al., 2022). By contrast, a more recent systematic review has concluded that sedentary behavior is typically associated with compromised integrity of brain structure in a broadly distributed set of regions (Zou et al., 2024). In parallel, the neural correlates of mental well-being have been the subject of intensive investigation in neuroimaging (de Vries, van de Weijer, & Bartels, 2023; Gatt et al., 2018; King, 2019; Lewis, Kanai, Rees, & Bates, 2014; Matsunaga et al., 2016; Sato et al., 2015). For instance, life satisfaction, reflecting an individual’s general evaluation of their overall quality of life, has shown both positive and negative associations with regional gray matter volume (Kong et al., 2015) as well as a negative association with cortical thickness (Zhu et al., 2018). Likewise, positive emotionality, reflecting an individual’s affective well-being, has been proved to be both positively and negatively associated with regional gray matter volume (Stretton, Schweizer, & Dalgleish, 2022).

Although the above-mentioned studies provide important information on the relationships between somatic movement, brain structures, and mental well-being, the majority are observational in nature. Due to the inherent limitations of observational studies such as residual confounding, information bias, and selection bias (Boyko, 2013; Yang et al., 2010), the causality of such associations remains undetermined. Common methods for interpreting causality include randomized controlled trials and Mendelian randomization (MR). However, randomized controlled trials suffer from limitations including high costs, high failure rates, and limited external validity (Heindel, Dieffenbach, Freeman, McGinigle, & Menard, 2022). Instead, MR constitutes a novel approach that utilizes genetic variants as instrumental variables (IVs) to infer causal relationships between exposures and outcomes. Benefiting from the randomness of natural genetic variation, MR circumvents the confounding factors commonly present in traditional observational studies, providing more reliable insights into causality (Richmond & Davey Smith, 2022). Moreover, the recent public availability of large-scale summary data from numerous genome-wide association studies (GWAS) renders MR a widely used tool that has attracted intense interest from researchers from different disciplines (Gagnon et al., 2024; Levin & Burgess, 2024; Tin & Köttgen, 2021).

In the present study, we aimed to disentangle the causal relationships among somatic movement, brain structures, and mental well-being. To achieve this goal, we applied a multi-stage MR framework to multi-source GWAS summary data. Building on previous literature, we hypothesized that somatic movement would be causally linked to mental well-being and brain structural changes, with the affected brain structures mediating the effects of somatic movement on mental well-being.

## Methods and materials

### Study overview

**Figure 1.**
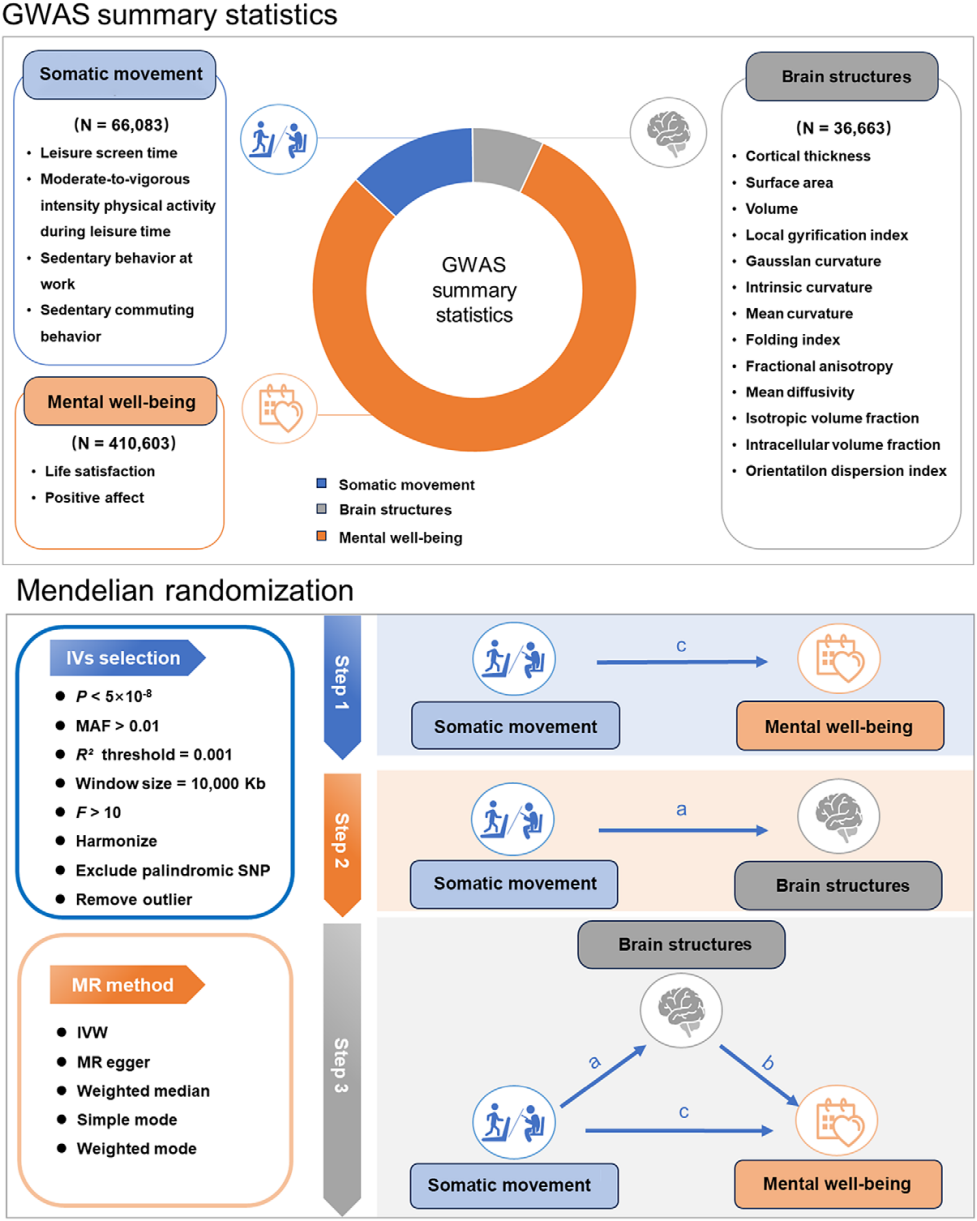
Overview of the multi-source GWAS data integration and the different levels of MR analyses. Abbreviations: GWAS, genome-wide association study; IV, instrumental variable; IVW, inverse variance weighted; MAF, minor allele frequency; MR, Mendelian randomization; SNP, single-nucleotide polymorphism.

We first assessed the causal associations between somatic movement and mental well-being using a two-sample MR, which utilized single-nucleotide polymorphisms (SNPs) derived from the summary-level GWAS data as instrumental variables to proxy for each exposure. We then performed a two-sample MR to investigate the causal effects of somatic movement on brain structures. Finally, we utilized a two-step MR analysis to assess whether brain structures play a causal role in mediating the pathway from somatic movement to mental well-being. An overview of the study is shown in Figure 1.

### Data sources

In this MR study, data sources of exposures, mediators, and outcomes were derived based on summary-level data from GWAS conducted in individuals of European ancestry (Supplementary Table 1).

#### Exposures

The genetic information of four somatic movement measures, assessed by self-reported questionnaires, was obtained through a GWAS meta-analysis of 51 studies of European populations, including leisure screen time (LST, *N* = 526,725), moderate-to-vigorous intensity physical activity during leisure time (MVPA, *N* = 606,820), sedentary behavior at work (SDW, *N* = 372,605), and sedentary commuting behavior (SDC, *N* = 159,606) (Wang et al., 2022).

#### Mediators

The genetic associations for brain structures were derived from a genome-wide association meta-analysis of 13 structural and diffusion magnetic resonance imaging-derived cortical phenotypes, measured globally and at 180 bilaterally averaged cortical regions in 36,663 individuals (Supplementary Table 2). These phenotypes included cortical thickness, surface area, gray matter volume, measures of folding, neurite density, and water diffusion (Warrier et al., 2023). This resulted in a total of 2,347 regional and global brain structures analyzed in the study.

#### Outcomes

Genetic variants for mental well-being indices including life satisfaction (LS, *N* = 80,852) and positive affect (PA, *N* = 80,852) were extracted from multivariate genome-wide analyses of the well-being spectrum (Baselmans et al., 2019).

### Genetic instruments selection

We firstly selected genome-wide significant SNPs for each trait with *P* < 5 × 10^−8^ for clumping. We then applied linkage disequilibrium (LD) analyses from the original GWAS for each trait, using a strict cut-off of *r^2^* < 0.001 and a window of 10,000 kb. Variants with minor allele frequency < 0.01 in the GWAS data set were removed, and we used the clump function within PLINK software to identify independent SNPs for each exposure (Purcell et al., 2007), using the 1000 Genomes European data as the reference. For all SNPs, we harmonized coding and non-coding alleles in the summary statistics of each GWAS. If no SNPs significant in exposure data could be found in outcome data, this portion of SNPs was removed, and no proxy SNPs were sought. We excluded SNPs whose *F*-statistic was <10 (a measure of the strength of these IVs) to avoid weak instrumental bias (Stephen Burgess & Thompson, 2011).

### Statistical analyses

#### Two-sample MR

Two-sample MR was performed to investigate if somatic movement causally affects mental well-being (c). We utilized the inverse variance weighted (IVW) method in the main analyses (Bowden & Holmes, 2019), since it is the most efficient analysis method with valid instrumental variables and could account for heterogeneity in the variant-specific causal estimates (Stephen Burgess et al., 2019). The *P* values from the IVW MR tests were adjusted using Bonferroni correction for multiple testing. To enhance the reliability of our results, we additionally used four MR methods (MR-Egger, weighted median, simple mode, and weighted mode) that make differing pleiotropy assumptions (Sanderson et al., 2022).

#### Mediation analysis

A two-step MR was performed to assess whether brain structures mediate the causal associations between somatic movement and mental well-being. The first step was to estimate the causal effects of somatic movement on brain structures (a) using two-sample MR, and the second step was to estimate the causal effects of brain structures on mental well-being (b) using two-sample MR. Then, the indirect effects of somatic movement on mental well-being through brain structures were evaluated using the product of coefficients method (*a* × *b*) (Carter et al., 2021). The 95% confidence intervals (CIs) were calculated using the RMediation package (Tofighi & MacKinnon, 2011). The mediation proportion was estimated by dividing the indirect effect (*a* × *b*) by the total effect (*c*).

#### Sensitivity analysis

We conducted several sensitivity analyses to evaluate the robustness of the MR results. First, we assessed horizontal pleiotropy using the MR-Egger method, which also provided a consistent estimate of the causal effect under the InSIDE (instrument strength independent of direct effect) assumption. The intercept represents the average pleiotropic effect across the genetic variants (the average direct effect of a variant with the outcome). If the intercept differed from zero (MR-Egger intercept *P*-value <0.05), there was evidence of horizontal pleiotropy (Stephen Burgess & Thompson, 2017). Additionally, we implemented the MR Pleiotropy RESidual Sum and OutLier (MR-PRESSO) (Verbanck, Chen, Neale, & Do, 2018) global test to further evaluate and address the influence of pleiotropy, with a *P* value <0.05 indicative of the presence of pleiotropy. Second, we assessed heterogeneity using Cochran’s *Q* statistic, and a *P* value of less than 0.05 indicated the presence of heterogeneity. Finally, the statistical power of the selected genetic tools was calculated using the online web tool (https://sb452.shinyapps.io/power/) according to the methodology described by Burgess (S. Burgess, 2014). Finally, we conducted a supplementary MRMix analysis to account for potential biases from sample overlap and weak instruments (Qi & Chatterjee, 2019).

#### Analytical tools

All MR analyses were conducted using R packages ‘TwoSampleMR’, ‘foreach’, ‘doParallel’, ‘ieugwasr’, ‘RadialMR’, ‘MRMix’, and ‘data.table’ in R software (version 4.3.2; the R Foundation for Statistical Computing, Vienna, Austria).

## Results

### Causal effects of somatic movement on mental well-being

**Figure 2.**
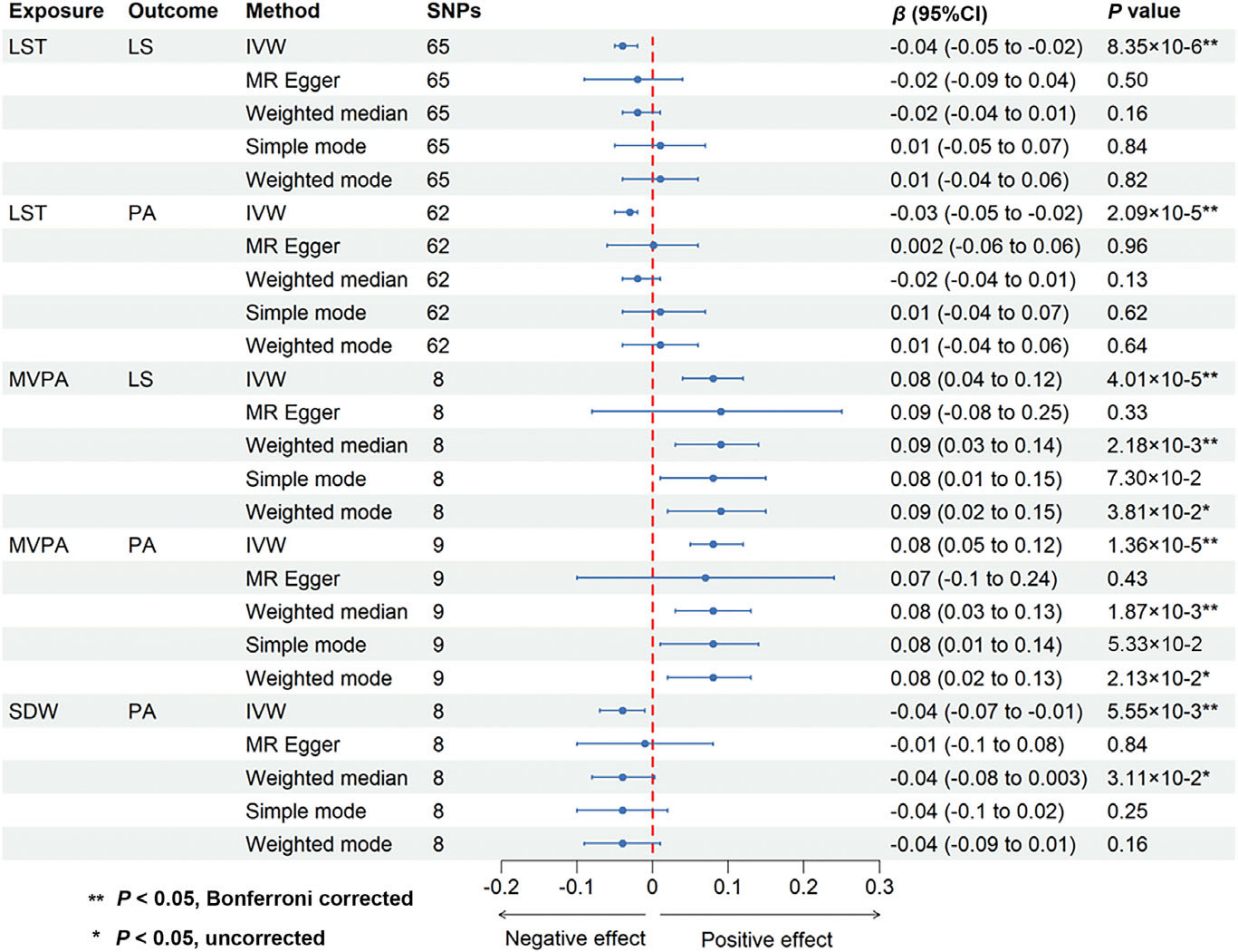
Causal effects of somatic movement on mental well-being. Abbreviations: CI, confidence interval; IVW, inverse variance weighted; LS, life satisfaction; LST, leisure screen time; MR, Mendelian randomization; MVPA, moderate-to-vigorous intensity physical activity during leisure time; PA, positive affect; SDW, sedentary behavior at work.

We conducted two-sample MR analyses to investigate the causal effects of somatic movement on mental well-being. The IVW method revealed that three somatic movement measures were causally associated with mental well-being (*P* < 0.05, Bonferroni corrected) (Figure 2 and Supplementary Table 3). Specifically, a 1-standard deviation (SD) increase in genetically predicted MVPA was causally associated with greater LS (*β* = 0.08 SD; 95% CI, 0.04–0.12; *P* = 4.01 × 10^−5^) and PA (*β* = 0.08 SD; 95% CI, 0.05–0.12; *P* = 1.36 × 10^−5^), while genetically predicted LST (per SD increase) was causally associated with lower LS (*β =* −0.04 SD; 95% CI, −0.05 to −0.02; *P* = 8.35 × 10^−6^) and PA (*β* = −0.03 SD; 95% CI, −0.05 to −0.02; *P* = 2.09 × 10^−5^). We also found that genetically predicted SDW (per SD increase) was causally associated with lower PA (*β* = −0.04 SD; 95% CI, −0.07 to −0.01; *P* = 5.55 × 10^−3^). The consistency of the IVW estimates was supported across multiple sensitivity analyses, including the absence of heterogeneity (all *P* for Cochran’s *Q* test >0.05) (Supplementary Table 4), strong instrument strength (*F*-statistics >10) (Supplementary Table 5) and statistical power (Supplementary Table 6), and the absence of substantial horizontal pleiotropy: non-significant MR-Egger intercepts and non-significant MR-PRESSO global tests where applicable (all *P* for Egger intercept and MR-PRESSO global test >0.05) (Supplementary Table 7), alongside highly consistent weighted median estimates (Supplementary Table 3).

### Causal effects of somatic movement on brain structures

**Figure 3.**
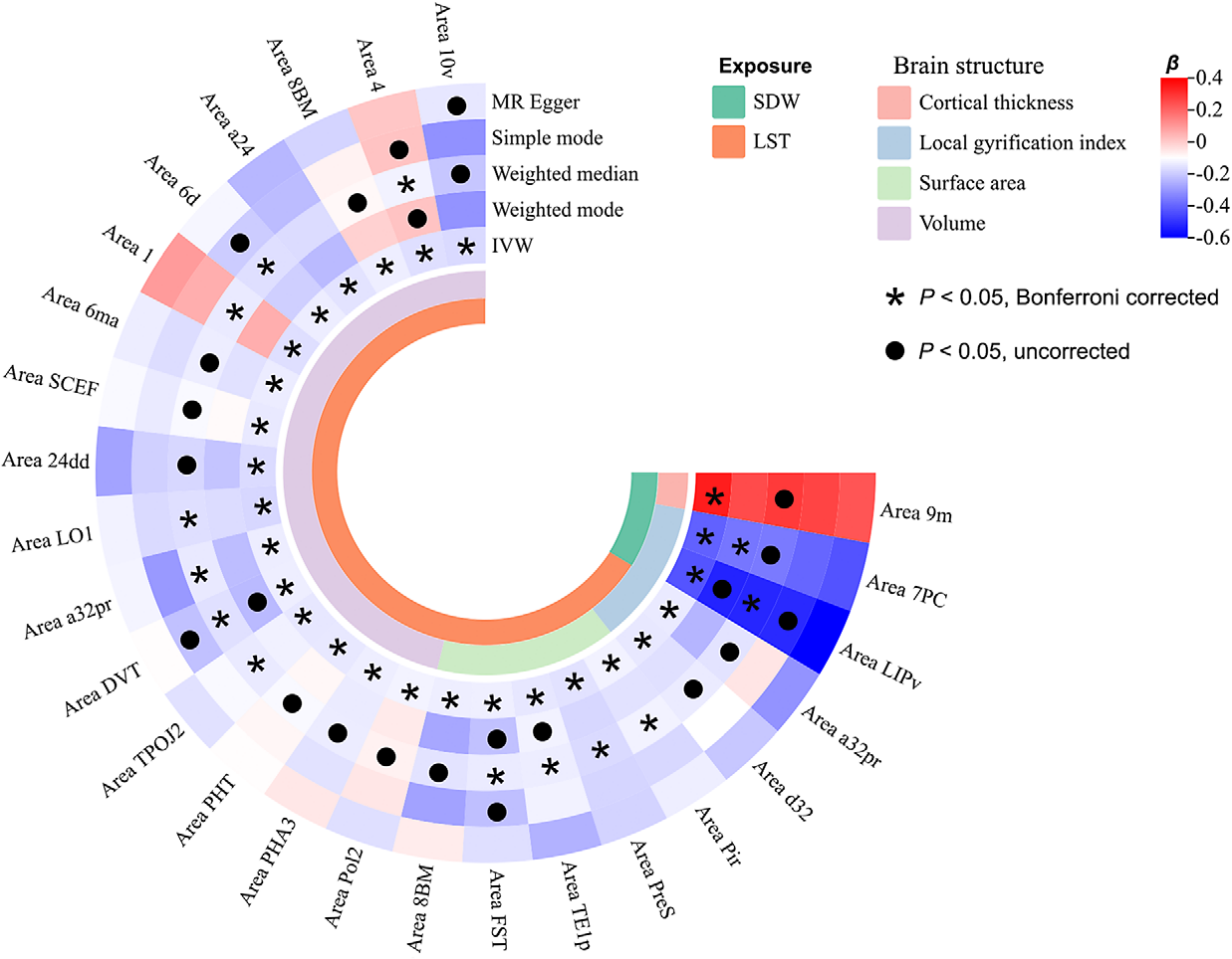
Causal effects of somatic movement on brain structures. Color represents *β* coefficient. Abbreviations: IVW, inverse variance weighted; LST, leisure screen time; MR, Mendelian randomization; SDW, sedentary behavior at work.

Two-sample MR analyses were also performed to explore the causal effects of somatic movement on brain structures. The IVW method revealed that LST and SDW were causally linked to changes of 26 brain structures (*P* < 0.05, Bonferroni corrected) (Figure 3 and Supplementary Table 8). Specifically, longer LST was causally linked to decreased volume of 16 cortical regions (Area 10v, Area 4, Area 8BM, Area a24, Area 6d, Area 1, Area 6ma, Area SCEF, Area 24dd, Area LO1, Area a32pr, Area DVT, Area TPOJ2, Area PHT, Area PHA3, and Area Pol2), decreased surface area of 5 regions (Area 8BM, Area FST, Area TE1p, Area PreS, and Area Pir), and decreased local gyrification index of 2 regions (Area a32pr and Area d32). More SDW was causally linked to increased cortical thickness of Area 9m and decreased local gyrification index of Area 7PC and Area LIPv. The consistency of the IVW estimates was supported across multiple sensitivity analyses, including the absence of heterogeneity (all *P* for Cochran’s *Q* test >0.05) (Supplementary Table 4), strong instrument strength (*F*-statistics >10) (Supplementary Table 5) and statistical power (Supplementary Table 6), and no detectable horizontal pleiotropy per MR-Egger and MR-PRESSO tests (all *P* for Egger intercept and MR-PRESSO global test >0.05) (Supplementary Table 7), with further confirmation from directionally aligned weighted median estimates (Supplementary Table 8).

### *Mediating the role of brain structure in the causal associations between somatic movement and mental* well-being

**Figure 4.**
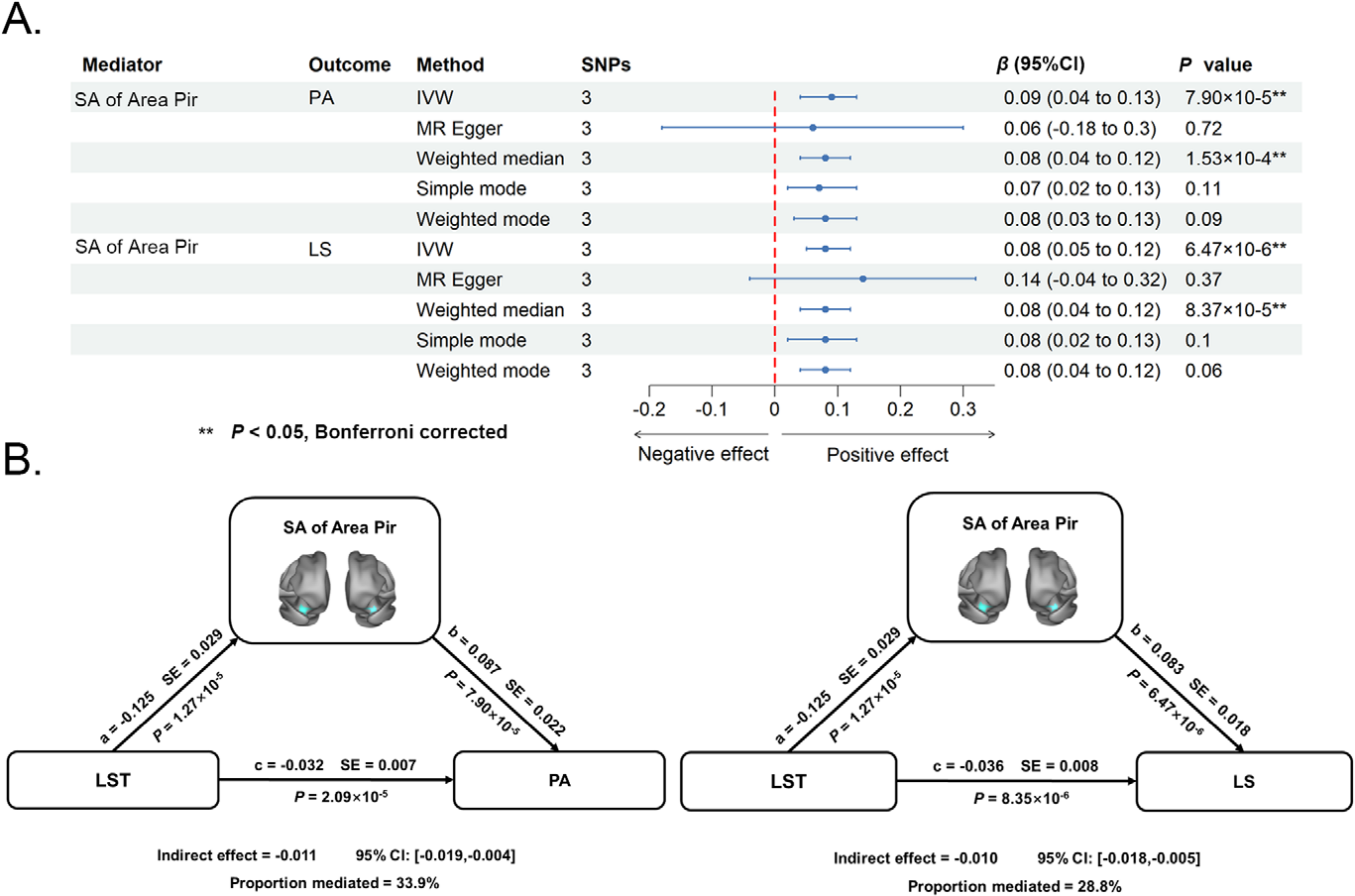
Mediating role of brain structure in the causal associations between somatic movement and mental well-being. (A) Causal effects of brain structure on mental well-being. (B) Two causal mediating pathways from somatic movement to mental well-being through brain structure. Abbreviations: CI, confidence interval; IVW, inverse variance weighted; LS, life satisfaction; LST, leisure screen time; MR, Mendelian randomization; PA, positive affect; Pir, piriform cortex; SA, surface area.

We adopted two-sample MR analyses to examine the causal effects of the above-identified 26 brain structures on mental well-being. As shown in Figure 4A and Supplementary Table 9, the IVW method revealed that genetically predicted surface area of the piriform cortex (per SD increase) was causally associated with greater LS (*β* = 0.08 SD; 95% CI, 0.05–0.12; *P* = 6.47 × 10^−6^) and PA (*β* = 0.09 SD; 95% CI, 0.04–0.13; *P* = 7.90 × 10^−5^) (*P* < 0.05, Bonferroni corrected). This also implies that decreased surface area of the piriform cortex was causally related to lower LS. All sensitivity analyses supported the direction and robustness of the primary IVW estimates. Specifically, we found no evidence of heterogeneity (all *P* for Cochran’s *Q* test >0.05) (Supplementary Table 4), strong instruments (*F*-statistics >10) (Supplementary Table 5) with sufficient power (Supplementary Table 6), and the absence of substantial horizontal pleiotropy: non-significant MR-Egger intercepts and non-significant MR-PRESSO global tests where applicable (all *P* for Egger intercept and MR-PRESSO global test >0.05) (Supplementary Table 7), alongside highly consistent weighted median estimates (Supplementary Table 9). Importantly, further mediation analyses showed that decreased surface area of the piriform cortex mediated the causal associations of longer LST with lower LS (indirect effect = −0.010; 95% CI, −0.018 to −0.005; proportion mediated = 28.8%) and PA (indirect effect = −0.011; 95% CI, −0.019 to −0.004; proportion mediated = 33.9%) (Figure 4B). Sensitivity analyses using the difference method yielded highly consistent estimates for both LS (indirect effect = −0.010; proportion mediated = 27.8%) and PA (indirect effect = −0.012, proportion mediated = 37.5%). The absolute differences in the proportion mediated between the product of coefficients and difference methods were 1.0% for LS and 3.6% for PA, further corroborating the robustness of our mediation findings against alternative estimation strategies. Nevertheless, the MRMix analysis indicated that accounting for potential biases from sample overlap and weak instruments influenced the effect estimates, although some associations remained significant. The complete results are provided in Supplementary Table 10.

## Discussion

We combined the multi-stage MR framework and multi-source GWAS summary data to investigate the causal relationships among somatic movement, brain structures, and mental well-being. Our data revealed that more physical activity (more MVPA) was causally associated with greater mental well-being (LS and PA), while more sedentary behavior (longer LST and more SDW) with lower mental well-being. With respect to brain structures, sedentary behavior was causally linked to decreased volume, surface area, and local gyrification index in distributed cortical regions. Remarkably, further mediation analysis demonstrated that decreased surface area of the piriform cortex mediated the causal associations between sedentary behavior and lower mental well-being. These findings not only complement and extend earlier reports on the associations of somatic movement with mental well-being and brain health by further resolving the causality but also help elucidate the neural mechanisms by which sedentary behavior adversely affects mental well-being.

### Causal associations between somatic movement and mental well-being

Earlier results from randomized controlled trials and prospective cohort studies suggest that physical activity, measured through either self-report or objective methods, can reduce the risk of mental illness, especially ameliorating depressive symptoms and enhancing population mental well-being (Chekroud et al., 2018; Harvey et al., 2018; Ibáñez Román et al., 2023; Singh et al., 2023). Besides, numerous studies have demonstrated a negative association between sedentary behavior and mental health (Ellingson et al., 2018; Hoare et al., 2016; Teno, Silva, & Júdice, 2024). Prolonged periods of inactivity have been shown to exacerbate depressive symptoms and increase the risk of depression (Huang et al., 2020). Correspondingly, the results presented here extend earlier findings by clarifying the causality of such associations. Previous studies have provided several possible mechanistic explanations for these findings. For example, physical activity improves depressive symptoms and overall mood though various neuromolecular mechanisms, including increased expression of neurotrophic factors, increased availability of serotonin and norepinephrine, regulation of hypothalamic–pituitary–adrenal axis activity, and hypocampal neurogenesis (Carl Ernst, 2006; Deng, Aimone, & Gage, 2010; Gujral, Aizenstein, Reynolds, Butters, & Erickson, 2017; Moyers & Hagger, 2023).

### Causal associations between somatic movement and brain structures

Our MR analyses demonstrated significant causal effects of sedentary behavior on compromised brain structures, adding to a growing body of literature suggesting the importance of sedentary behavior in brain health. The associations we observed between sedentary behavior and brain structures align with a previous study reporting negative impacts of sedentary behavior on cognitive performance and brain integrity (Zou et al., 2024). Our findings showed negative effects of LST on volume and surface areas of 21 brain regions, which are distributed across extensive cortical areas, primarily in the anterior cingulate and medial prefrontal cortex, insular and frontal opercular cortex, and medial and lateral temporal cortex. The anterior cingulate cortex and medial prefrontal cortex are involved in reward processing, emotional regulation, and processes related to episodic memory (Rolls, Deco, Huang, & Feng, 2023b). The medial temporal lobe is a crucial region for cognitive functions in the brain (Rusinek et al., 2003). Meanwhile, the insula and frontal opercular cortex play critical roles in cognitive control, behavioral regulation, and emotional processing (Dixon, Thiruchselvam, Todd, & Christoff, 2017; R. Zhang, Deng, & Xiao, 2024). Therefore, disrupted cortical structures of these areas could theoretically impair cognitive efficiency and motor-sensory integration, which is consistent with previous research emphasizing the detrimental effects of prolonged screen exposure on brain health (Eikelboom, Ciccarelli, Tadros, & Manwell, 2022; Neophytou, Manwell, & Eikelboom, 2019). The negative effects of LST on LGI of Area a32pr and d32 as well as of SDW on LGI of Area 7PC and LIPv suggest the potential of sedentary behavior to alter the complex folding patterns of the brain. LGI, an indicator closely related to primary neuroconnectivity, is often considered a neurodevelopmental marker reflecting surface complexity and cortical connectivity in early neurodevelopment (R. Dauvermann et al., 2012). Studies have supported a mechanistic link between mental disorders and abnormal cortical folding (Choi et al., 2020; Long et al., 2020; Sanfelici et al., 2022; Sasabayashi, Takahashi, Takayanagi, & Suzuki, 2021; Y. Zhang et al., 2023). Interestingly, our study reported a positive effect SDW on cortical thickness of the prefrontal area 9m. While seemingly counterintuitive, this finding aligns with frameworks of neural compensation or cognitive reserve (Cabeza et al., 2018; Reuter-Lorenz & Park, 2014). The prefrontal cortex is a key site for such compensatory processes (Di Tella et al., 2021; Ren et al., 2025), and it is plausible that prolonged, cognitively demanding sedentary work (e.g. computer-based tasks, data analysis) promotes compensatory upregulation or neural scaffolding, manifesting as greater cortical thickness in area 9m. Alternatively, this association may reflect the established principle that different sedentary behaviors have divergent links to cognition (Bakrania et al., 2018; Mellow et al., 2021; Wanders et al., 2021). Large-scale studies show that while TV viewing and driving are inversely associated with cognitive function, cognitively engaging activities like computer use are positively associated with it (Bakrania et al., 2018). Thus, our finding may indicate that sedentary time in our cohort is predominantly of a cognitively vitalizing nature, which could promote neural scaffolding and cortical integrity as a protective mechanism. Future longitudinal studies with detailed assessments of sedentary behavior content are needed to elucidate the underlying mechanisms.

### Causal mediating pathways from somatic movement to mental well-being through brain structure

Our results indicated that the negative effects of LST on mental well-being were partially mediated by decreased surface area of the piriform cortex. Specifically, longer LST contributed to decreased surface area of the piriform cortex, which in turn led to diminished LS and attenuated PA. Characterizing the mediating role of the piriform cortex in accounting for the causal associations between sedentary behavior and mental well-being represents the most important contribution of the present study. Sedentary behavior has frequently been reported to be associated with changes in brain structures (Zou et al., 2024). For instance, some observational studies have demonstrated that screen-based sedentary behavior, such as watching television, appears to be unfavorably linked to certain measures of brain structures in children and adolescents (Takeuchi et al., 2018; Zavala-Crichton et al., 2020). In addition, cross-sectional studies have found that long-term sedentary behavior is associated with decreased medial temporal cortex thickness (Ginsberg et al., 2018) and decreased white matter volume (Arnardottir et al., 2016) in older adults. Despite these promising results, the effects of sedentary behavior on the piriform cortex have remained undiscovered, which may be due to the fact that most previous studies have focused on one single imaging modality. The current observation of such effect may profit from the utilization of a comprehensive set of neuroimaging modalities that can capture both morphological and microstructural characteristics of the cerebral cortex.

The piriform cortex has extensive connections with key limbic and cortical regions, including the hippocampus, amygdala, anterior insula, and medial prefrontal cortex (Kaur et al., 2025; Manahan-Vaughan & Strauch, 2020; Menelaou et al., 2024; Rolls, Deco, Huang, & Feng, 2023a), some of which are key nodes of the Salience Network (SN) and the Default Mode Network (DMN) (Ince et al., 2025; Jobson, Hase, Clarkson, & Kalaria, 2021; Menon, 2023). This foundational architecture positions it as a potential interface for integrating sensory information and influencing large-scale network dynamics. A growing body of functional neuroimaging evidence now substantiates this role. For instance, a recent study demonstrated that targeted electrical stimulation of the olfactory nerve not only boosted functional connectivity between the piriform cortex and the SN but also effectively weakened the hyperconnectivity between the SN and DMN – a pattern characteristic of depression (Heller et al., 2025). Furthermore, animal research has unraveled that the piriform cortex to prelimbic cortex loop mediates empathic social approach behavior in female mice, while the piriform cortex to medial amygdala loop mediates empathic self-grooming behavior in male mice (Fang et al., 2024). Although these precise circuit-level mechanisms remain beyond the interpretative scope of contemporary human neuroimaging, they collectively position the piriform cortex as an integral node within the distributed network governing social-affective behaviors. Building upon the aforementioned findings, we theorize how prolonged sedentary behavior might disrupt this delicate network balance. It is plausible that such behavior generates subtle but persistent interoceptive cues (e.g. muscular discomfort or reduced cardiopulmonary feedback) (Khalsa et al., 2018), which are known to be processed by the SN (Chong, Ng, Lee, & Zhou, 2016). Chronic exposure to such aberrant interoceptive signaling may ultimately lead to structural degradation of key circuit hubs, including the piriform cortex, which serves as a convergence zone for somatic and visceral information (Zhou, Lane, Cooper, Kahnt, & Zelano, 2019). The observed reduction in piriform cortex surface area in our study may thus represent a neuroanatomical signature of this maladaptive process. This structural alteration likely impairs the piriform cortex’s capacity to function as an effective interface. A compromised piriform cortex would be less able to facilitate the SN’s appropriate response to stimuli and to help suppress the self-referential activity of the DMN, as evidenced by the olfactory stimulation study (Heller et al., 2025). Consequently, the brain may become trapped in a state characterized by hypoactive SN and hyperactive DMN, which represents a neural signature characteristic of depression (Bodurka et al., 2016; Koeppel, Herrmann, Weidner, Linn, & Croy, 2021; Manoliu et al., 2014). Subjectively, this manifests as anhedonia, escalating self-focused rumination, and diminished positive affect (Disner, Beevers, Haigh, & Beck, 2011). These manifestations align precisely with the detrimental effects of sedentary behavior on mental well-being in our study.

When interpreting the aforementioned findings, a crucial consideration is the inherent heterogeneity of the ‘leisure screen time’ construct. The measurement approach employed in this study combines qualitatively distinct activities (such as passive TV watching and interactive video gaming or reading) into a composite metric. Existing evidence indicates that different types of leisure screen time have divergent links to mental well-being through distinct mechanisms (Sanders et al., 2023; Sanders, Parker, del Pozo-Cruz, Noetel, & Lonsdale, 2019; Santos et al., 2023). For instance, passive viewing may be more strongly associated with reduced cognitive engagement, whereas certain interactive uses could be linked to social pressure or cognitive stimulation (Bakrania et al., 2018; Mellow et al., 2021; Wanders et al., 2021). Consequently, the overall association observed in our study may be primarily driven by certain passive types of screen behaviors, while potential neutral or even positive effects of other types could have been obscured. This aggregation effect is critical for understanding the complex relationship between sedentary behavior and mental well-being.

There are some limitations to our study. First, our MR analyses included GWAS data sets from only individuals of European descent, which may limit the generalizability of the findings due to ethnic and regional factors. This is a critical consideration because genetic discoveries in one population may not translate directly to others due to differences in LD patterns, allele frequencies, and gene–environment interactions (Martin et al., 2019). Future research should prioritize the integration of diverse, multi-ancestry data sets and develop robust methods for cross-population causal inference, such as trans-ethnic MR (Hou, Wu, Yuan, Xue, & Li, 2025; Wojcik et al., 2019). Second, the somatic movement measures used in this study are self-reported and lacks objectivity, which may be influenced by bias in participant mood, memory inaccuracy, and social desirability (Prince et al., 2008). Future studies should therefore employ multi-method assessments. Specifically, integrating device-based objective measures (e.g. accelerometry) with self-reported data would help to validate findings, mitigate shared method bias, and provide a more holistic understanding of somatic movement across a wider range of behaviors and patterns. Third, the inclusion of UK Biobank individuals in exposure, mediator, and outcome data sets may introduce sample overlap bias, potentially causing biased effect estimates. However, this bias might have been mitigated, given the sufficient strength of the genetic instrumental variables (*F* statistic >10) (Stephen Burgess, Davies, & Thompson, 2016). Our MRMix analysis indicated that accounting for potential biases from sample overlap and weak instruments influenced the effect estimates. Therefore, our causal estimates should be considered preliminary, and future verification in completely independent samples is required. Fourth, the reliance on a composite ‘leisure screen time’ measure that combines passive and interactive activities may obscure potential differential effects and preclude insights into the unique implications of specific types of screen use. Future studies should adopt more nuanced measurement tools to separately capture different types of sedentary screen behaviors, thereby more clearly elucidating their independent associations with mental well-being. Fifth, a further limitation of our study is the direct removal of SNPs unavailable in the outcome data sets, which may lead to loss of genetic information. However, our analysis demonstrated that although the removal of missing SNPs resulted in a reduction in the variance explained, the *F*-statistics remained highly stable across all analyses (Supplementary Table 11). Future studies with better-matched genomic coverage should compare our removal-based approach with the proxy SNP method (*r* ^2^ ≥ 0.8) to provide valuable empirical evidence and further validate the robustness of their conclusions. Finally, this study employed Bonferroni correction to prioritize specificity, which may have excluded brain structures with weak individual effects but significant joint effects. Supplementary analyses showed that, although the marginally significant structures exhibited significant joint effects, the original 26 structures captured the vast majority of the total effect size (detailed in the Appendix), supporting the robustness of our core findings. Future studies could incorporate network analyses or whole-brain approaches to further elucidate the distributed mechanisms through which somatic movements influence mental well-being via brain structures.

In conclusion, we integrated the multi-stage MR framework and multi-source GWAS summary data to examine the causal relationships among somatic movement, brain structures, and mental well-being. Our results suggest that more physical activity and less sedentary behavior contribute to mental well-being and brain health. Importantly, we establish a potential pathway whereby structural integrity of the piriform cortex mediates the adverse effects of sedentary behavior on mental well-being. More broadly, the identified piriform cortex may serve as a promising noninvasive neuromodulation target for promoting mental well-being in individuals who are frequently exposed to sedentary behavior.

## Supporting information

10.1017/S0033291726104097.sm001 Guo et al. supplementary material 1 Guo et al. supplementary material

10.1017/S0033291726104097.sm002 Guo et al. supplementary material 2 Guo et al. supplementary material

## Data Availability

All GWAS summary statistics analyzed in this study are publicly available as shown in Supplementary Table 1 and can be downloaded by qualified researchers. The GWAS data for somatic movement traits can be obtained from the GWAS Catalogue (https://www.ebi.ac.uk/gwas/downloads/summary-statistics, GCP ID: GCP000358). The GWAS data for brain structure phenotypes are available for access here: https://portal.camide.cam.ac.uk/overview/483. The summary statistics from the genome-wide association meta-analyses for life satisfaction and positive affect have been deposited in the GWAS Catalogue under accession codes GCST007337 and GCST007338, respectively.

## Supplementary material

The supplementary material for this article can be found at http://doi.org/10.1017/S0033291726104097.
