# Supplementary material for: Causal relationships between somatic movement, brain structures, and mental well-being: A multi-stage Mendelian randomization study
Source: Psychol Med. 2026 Apr 10;56:e102. doi: 10.1017/S0033291726104097 (PMC13079230; doi:10.1017/S0033291726104097)
Supplement: Guo et al. supplementary material 1 — Guo et al. supplementary material [file S0033291726104097sup001.docx]

**Appendix**

**Sensitivity Analyses for Brain Structure Selection**

**Rationale for Supplementary Analyses**

Our primary analysis employed Bonferroni correction to identify brain structures significantly associated with somatic movements, prioritizing specificity to ensure valid causal inference. However, we acknowledge that this stringent approach might exclude structures with weak individual effects that could contribute meaningfully in joint analyses. To address this potential selection bias and evaluate the robustness of our findings, we conducted comprehensive sensitivity analyses using relaxed screening thresholds and polygenic risk score (PRS)-based MR (Lee, Stapleton, MacGregor, & Mackey, 2025; Xu et al., 2025; Zhang et al., 2025).

**Methods**

**FDR Screening**

We expanded the instrumental variable selection using the Benjamini-Hochberg false discovery rate (FDR) correction (*P* < 0.05), identifying 154 brain structures associated with somatic movements, including the original 26 Bonferroni-significant regions and additional 128 marginally significant structures.

**Multivariable MR**

For each of the 128 additionally included structures, we performed multivariable Mendelian randomization (MVMR) with somatic movements and the respective brain structure as joint exposures and mental well-being as the outcome (Burgess & Thompson, 2015; Sanderson, 2021). This assessed whether these marginally significant structures exhibited independent causal effects when accounting for somatic movements.

**PRS-based MR Analysis**

We constructed PRS analysis for two sets of brain structures: (i) the original 26 Bonferroni-significant structures, and (ii) the 128 additionally included structures. PRS-based MR was conducted using IVW to estimate the joint causal effects on mental well-being.

**Results**

**MVMR Heterogeneity**

MVMR analysis of the 128 marginally significant structures revealed that although some structures exhibited nominal significance (*P* < 0.05), nearly all models displayed highly significant Cochran's *Q* statistics (*P* < 0.0001), indicating substantial heterogeneity among instrumental variables (Supplementary Table 12). This heterogeneity likely arose from the relaxed screening threshold, which may have introduced weakly associated instruments or those with stronger horizontal pleiotropy, thereby compromising the stability of causal effect estimates for individual structures.

**PRS-based MR Comparison**

The results demonstrated significant joint effects of the 128 FDR-identified structures on both mental health outcomes (LS: *β* = -0.0297, *P* = 5.0 × 10^⁻5^; PA: *β* = -0.0321, *P* = 6.5 × 10⁻⁷), whereas the original 26 Bonferroni-significant structures captured 90.9% and 76.3% of the total effect sizes, respectively (Supplementary Table 13). These findings suggest that although stringent screening may have missed some weak signals, the originally identified 26 brain structures captured the vast majority of the causal signal, further supporting the robustness of our core conclusions.

**Conclusion**

Our conservative screening strategy, while potentially excluding weakly associated structures, ensured the selection of genetic instruments satisfying MR core assumptions. Future studies could incorporate network analyses or whole-brain approaches to further elucidate the distributed mechanisms through which somatic movements influence mental well-being via brain structures.

**Appendix References**

Burgess, S., & Thompson, S. G. (2015). Multivariable Mendelian Randomization: The Use of Pleiotropic Genetic Variants to Estimate Causal Effects. *American Journal of Epidemiology, 181*(4), 251-260. doi:10.1093/aje/kwu283

Lee, S. S.-Y., Stapleton, F., MacGregor, S., & Mackey, D. A. (2025). Genome-wide association studies, Polygenic Risk Scores and Mendelian randomisation: an overview of common genetic epidemiology methods for ophthalmic clinicians. *British Journal of Ophthalmology, 109*(4), 433-441. doi:10.1136/bjo-2024-326554

Sanderson, E. (2021). Multivariable Mendelian Randomization and Mediation. *Cold Spring Harbor Perspectives in Medicine, 11*(2). doi:10.1101/cshperspect.a038984

Xu, L., Zhou, G., Jiang, W., Zhang, H., Dong, Y., Guan, L., & Zhao, H. (2025). JointPRS: A data-adaptive framework for multi-population genetic risk prediction incorporating genetic correlation. *Nature Communications, 16*(1). doi:10.1038/s41467-025-59243-x

Zhang, H., Long, X., Niu, G., Shi, W., Zhao, Z., Feng, D., . . . Wu, Y. (2025). Genetic insights into lipid traits and atherosclerosis risk: a Mendelian randomization and polygenic risk score analysis. *International Journal of Surgery, 111*(10), 6802-6815. doi:10.1097/js9.0000000000002869
